# Neighbouring group participation hindered by force as a molecular design for covalent catch bonds

**DOI:** 10.1038/s41467-026-73312-9

**Published:** 2026-05-22

**Authors:** Soumabrata Majumdar, Diederik van Luijk, Martijn M. van Galen, Pascal Vermeeren, Trevor A. Hamlin, F. Matthias Bickelhaupt, Joris H. B. Sprakel, Rolf A. T. M. van Benthem, Johan P. A. Heuts, Rint P. Sijbesma

**Affiliations:** 1 Department of Chemical Engineering & Chemistry and Institute for Complex Molecular Systems, Eindhoven University of Technology, Eindhoven, The Netherlands; 2 Physical Chemistry and Soft Matter, Wageningen University and Research, Wageningen, The Netherlands; 3 Laboratory of Biochemistry, Wageningen University and Research, Wageningen, The Netherlands; 4 Department of Chemistry and Pharmaceutical Sciences, Amsterdam Institute of Molecular and Life Sciences (AIMMS), Vrije Universiteit Amsterdam, Amsterdam, The Netherlands; 5 Institute of Molecules and Materials (IMM), Radboud University, Nijmegen, The Netherlands; 6 Department of Chemical Sciences, University of Johannesburg, Johannesburg, South Africa; 7 Department of Chemistry & Chemical Engineering, Laboratory of Physical Chemistry and Center for Multiscale Electron Microscopy, Eindhoven University of Technology, Eindhoven, The Netherlands; 8 Energy Transition Center Amsterdam, Amsterdam, The Netherlands

**Keywords:** Polymers, Computational chemistry, Mechanical properties

## Abstract

Catch bonds—dynamic molecular interactions whose lifetimes increase under mechanical load—are central to biological mechanotransduction but remain challenging to replicate synthetically. Here, we report a covalent catch-bonding mechanism in a low-molecular-weight motif based on hydroxyethyl phosphate (HEP) triesters. Our design uses force-mediated inhibition of a neighboring group participation (NGP) pathway: mechanical tension suppresses intramolecular assistance, thereby increasing the reaction barrier and prolonging bond lifetimes. Density Functional Theory calculations confirm that tensile force hinders the geometric contraction required for NGP, providing a mechanistic basis for catch-bond behaviour. Single-molecule force spectroscopy reveals that HEP triester lifetimes increase over threefold at 400 pN. This work establishes a molecular mechanism for engineering covalent catch bonds, offering opportunities to design force-responsive polymer networks. By translating a biological concept into a synthetic framework, our findings open new avenues for adaptive materials and mechanochemical sensing.

## Abstract (web summary)

Catch bonds—dynamic molecular interactions whose lifetimes increase under mechanical load—are central to biological mechanotransduction but remain challenging to replicate synthetically. Here, the authors report a covalent catch-bonding mechanism in a low-molecular-weight motif based on hydroxyethyl phosphate triesters.

## Introduction

Controlling the stability of reversible molecular bonds under mechanical force provides a powerful strategy for tuning the mechanical response of (macro)molecular systems. In biological contexts, reversible bonds often involve protein–ligand complexes that weaken under tensile stress, resulting in reduced lifetimes under load. In contrast, a distinct class of interactions—catch bonds—exhibits the opposite behavior: their lifetimes increase when subjected to tension^1^. Catch bonds play critical roles in physiological processes such as blood clotting^2^ and cell adhesion under flow^2^. Recent studies have further demonstrated that biological catch bonds can suppress crack initiation in soft biomimetic materials^3^.

Biological catch bonds rely on force-induced conformational changes in proteins, which are large, high-molecular-weight functional units. Synthetic strategies reported to date mimic this behavior using significantly smaller functional units^4,5^. Notably, synthetic catch bonds based on DNA base-pair interactions have been experimentally realized, opening avenues for designing a new generation of force-responsive biomimetic materials^4^.

In synthetic polymers, covalent catch bonds could offer similar control over mechanical properties as their non-covalent counterparts, while providing superior strength and requiring smaller force-responsive motifs. However, computational and experimental studies indicate that most covalent bonds behave as “slip bonds,” with lifetimes decreasing as applied force increases^6^. This trend has been confirmed for various functional groups through single-molecule force spectroscopy^5,7,8^ and reactivity studies involving strained cyclic molecules^9–12^. Nevertheless, exceptions exist: siloxane Si–O bonds^12^, Diels–Alder cycloadducts^13,14^, ladderanes^15^, and ketene dimers^16^ have been reported or predicted to exhibit catch-bond-like behavior under specific conditions.

As these examples illustrate, covalent catch bonds described so far are structurally diverse, precluding the formulation of a common design principle. Here, we propose a molecular mechanism for covalent catch bonding when bond dissociation takes place via neighboring group participation (NGP). We validate this concept using a hydroxyethyl phosphate (HEP) triester linkage embedded in a polymer and suggest that the approach holds promise to be generalized to other NGP-based systems with appropriate modifications.

**Fig. 1:**
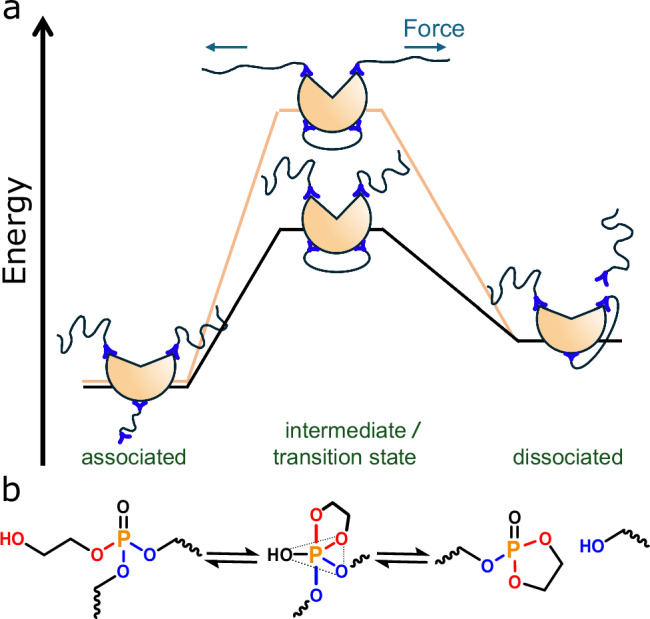
Molecular mechanism for covalent catch bond design. **a** Design of a covalent catch bond based on hindering the neighboring group participation (NGP) when force is applied. Catch bonding involves NGP that requires reduction in separation of the polymer attachment points when proceeding from the associated to the intermediate/transition state. Under force, the contraction increases the reaction barrier, slowing down the dissociation. **b** Reaction via NGP in a 2-hydroxyethyl phosphate triester representing the catch bond design in (**a**). Reduction of the O-P-O bond angle between polymer attachment points (wiggly lines) during dissociation increases the reaction barrier under force.

NGP is well known to accelerate chemical reactions by several orders of magnitude^17^. We hypothesized that a molecular moiety undergoing NGP-mediated bond dissociation could function as a catch bond if NGP is sterically or geometrically hindered under tension. Mechanistically, this scenario arises when NGP requires a reduction in the distance between polymer attachment points during the transition from the associated to the dissociated state. Under tensile load, this geometric contraction incurs an additional energetic penalty, thereby increasing the activation barrier and slowing the reaction (Fig. 1a). This behavior aligns with the classical one-state, two-path model^18^, where the catch pathway involves NGP-mediated cleavage and the slip pathway proceeds via alternative high-force routes such as homolytic or heterolytic bond scission.

To test this hypothesis, we identified a 2-hydroxyethyl phosphate (HEP) triester linkage as a suitable candidate. In this system, transesterification is facilitated by intramolecular participation of the hydroxy group, analogous to the mechanism that accelerates RNA hydrolysis by more than five orders of magnitude relative to DNA^19,20^. During the transition from reactant to transition state, the phosphorus center undergoes a geometry change from tetrahedral to trigonal bipyramidal, reducing the O–P–O bond angle (Fig. 1b). Consequently, polymer chains attached to the phosphorous center move closer together, opposing an applied tensile force. This opposition necessitates additional work to achieve the transition state under elongational stress, slowing down the reaction. Based on this mechanistic framework, we hypothesized that HEP triesters embedded in polymers will exhibit catch-bond behavior.

## Results and discussion

**Fig. 2:**
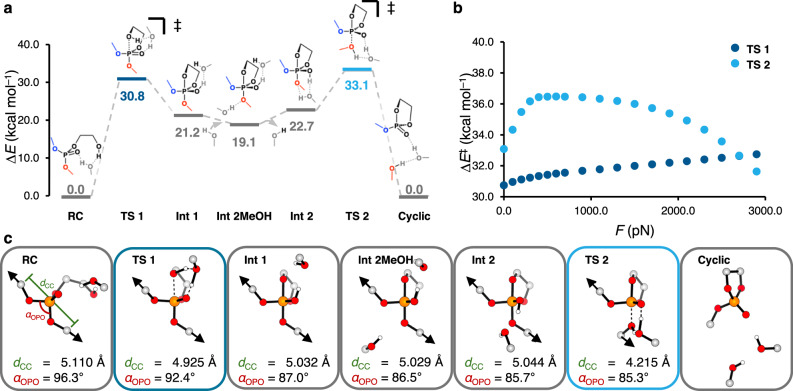
Force-free and force-dependent DFT calculations of the ring-closing reaction of DMHEP. **a** Potential energy diagram for ring closing reaction of a model HEP species modeled with one explicit methanol solvent molecule (two explicit methanol solvent molecules for intermediate **Int 2MeOH**) in the absence of an externally simulated force; **b** activation energies of transition states **TS 1** and **TS 2** with respect to reactant complex **RC** as a function of the externally simulated force; **c** stationary point structures with key geometrical information in the absence of an externally simulated force at ZORA-OLYP/TZ2P. Aliphatic hydrogen atoms are omitted for clarity.

Density functional theory (DFT) calculations were carried out to study the reaction mechanism of the ring-closing step of phosphate transesterification using the AMS software package^21–23^ at the ZORA-OLYP/TZ2P level of theory^24–27^. An archetypal model system of the polymer, namely, dimethyl(hydroxyethyl) phosphate (**DMHEP**), was employed to provide physical insight into the factors controlling the S_N_2@P addition-elimination transformation^28,29^. Further computational details are described in the methods section. An explicit methanol solvent molecule was included in the calculations to equip the model system with the physics of a potential solvent-assisted proton transfer step^30,31^. During the exchange reaction, from the reactant complex **RC** to the cyclic product (**Cyclic**), the reaction pathway crosses two transition states, denoted **TS 1** and **TS 2**, which are connected by a shallow potential energy surface associated with three trigonal bipyramidal intermediates (Fig. 2a). It is in these three intermediates where the coordination and dissociation of a second explicit methanol solvent molecule take place, from **Int 1** to **Int 2MeOH** to **Int 2**, to facilitate the formation of the cyclic product in the second reaction step. A careful examination of the stationary point energies and structures reveals that (i) both transition states closely resemble the trigonal bipyramidal nature of the intermediates, and (ii) **TS 2** is the rate-limiting step in the overall ring-closing reaction (Fig. 2a and c).

Introducing a constant external force pulling on the two methyl groups^32^, indicated by black arrows in Fig. 2c, drastically destabilizes the transition states (Fig. 2b). Thus, **TS 1,** **TS 2**, as well as the intermediates (Supplementary Table 1) rise in energy relative to the **RC**, as the direction of the external force vector penalizes the trigonal bipyramidal-like geometry of these pentacoordinate phosphorus stationary points (Fig. 2b). Similar force-dependent reaction barriers have been reported for mechanochemical reactions involving stationary points that have a trigonal bipyramidal geometry^12^. When the applied external force exceeds a magnitude beyond 500 pN, **TS 2** becomes stabilized, because the applied force facilitates the cleavage of the MeOH leaving group. At external forces above 2500 pN the energy of **TS 2** drops below **TS 1**, thus rendering **TS 1** rate-limiting. The relevance of these strongly applied external forces to dynamic polymer behavior, however, is uncertain because irreversible scission of C–C and C–O bonds in a polymer may be the dominant dissociative reaction at forces above 2500 pN^6^. Overall, the bond dissociation reaction proceeds with a higher reaction energy barrier than the force-free reaction up to 2.5 nN due to an increase of both the **TS 1** and **TS 2** barriers. The effect is strongest at forces below 500 pN, where **TS 2** is rate-limiting. Thus, our quantum chemical analyses predict catch bond behavior in HEP triesters.

**Fig. 3:**
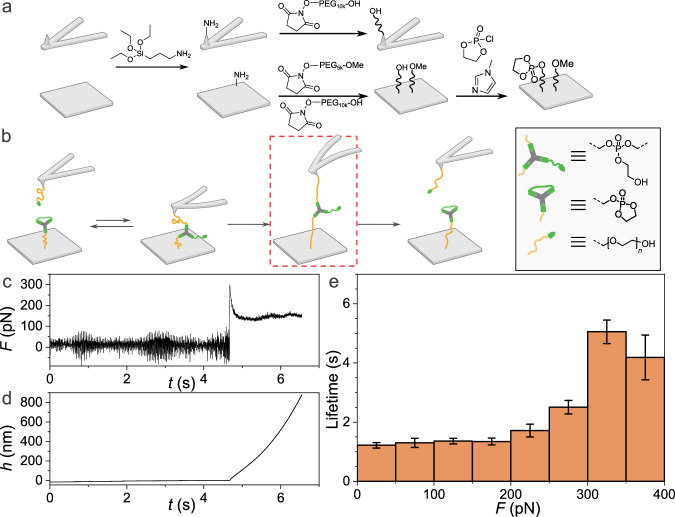
SMFS experiments to probe the force-dependent ring-closing reaction of HEP triesters. **a** cartoon of the functionalization of the silicon wafer and silicon nitride AFM tip used for the SMFS measurements; **b** cartoon of SMFS experiment in which a HEP triester is formed in situ between tip and surface, followed by retraction to a constant force until the bond is broken spontaneously; **c** example of measured restoring force between surface and tip in an experiment with a single event; **d** measured height of the AFM tip in the same experiment; **e** binned exponential lifetime for various forces extracted from SMFS measurements. Error bars represent the standard error of the fitted lifetime parameter. Cartoons of wafers and AFM tips were made by the ICMS Animation Studio.

The presence of a force-decelerated reaction pathway was investigated experimentally using single-molecule force spectroscopy (SMFS) by probing the force-dependent lifetime of a HEP triester. This was accomplished using a polymer-functionalized silicon wafer substrate and Si_4_N_3_ atomic force microscopy (AFM) tip. The substrate was densely functionalized with methoxy-terminated PEG chains, interspersed with 5% PEG chains with cyclic ethylene phosphate triester groups (Fig. 3a). AFM tips were functionalized with hydroxy-terminated PEG chains, allowing the HEP triester bond to be formed and broken between the tip and surface in situ as shown schematically in Fig. 3b. The tip was briefly held in contact with the surface in anhydrous DMF at 60 °C and then retracted to maintain a constant tensile force for up to 30 s. In that time, the backward reaction involving intramolecular transesterification may occur, reforming the five-membered ring and severing the connection between tip and wafer. This is observed as a sudden loss of tensile force and an increase in tip height. (Fig. 3 c, d). When exactly one independent dissociation event was observed in a measurement, it was considered a valid single event, and the restoring force and bond lifetime were recorded. Excluding very short individual bond lifetimes below 0.2 s, which may be caused by non-covalent interactions between the tip and surface (Supplementary methods and data for SMFS experiments). Events were combined in 50 pN wide bins and the fraction of intact bonds vs. time within a bin was fitted to an exponential to extract the lifetime. These lifetimes are roughly constant at 1 s up to a force of 200 pN and increase to approximately 4 s at 400 pN (Fig. 3e). This observation agrees with the computed increase in reaction barrier (Fig. 2b). Due to the challenging conditions for SMFS measurements, insufficient dissociation events were observed above 400 pN to extract reliable lifetimes.

In conclusion, we demonstrate that a hydroxyethyl phosphate (HEP) triester linkage in a polymer acts as a molecular-scale catch bond through a force-decelerated NGP-mediated transesterification reaction. Quantum chemical calculations predict that the applied force raises the reaction barrier for bond dissociation. This finding is qualitatively supported by single-molecule force spectroscopy, which shows a more than threefold increase in bond lifetime between 200 and 400 pN. Given the relevance of HEP ester chemistry in RNA and dynamic polymers, this discovery is expected to stimulate research on force-adaptive behavior in other HEP-containing systems. Moreover, the simplicity of the underlying principle—suppression of NGP under tension—suggests that this concept could be extended to other NGP-mediated reactions. Although linking single-molecule force-dependent reactivity to macroscopic material properties remains a challenge, covalent catch bonds offer a promising route to mechanically adaptive materials that resist fracture, enable controlled degradation under load, or allow reprocessing under tailored conditions.

## Methods

### Materials

All reagents and solvents were used as obtained without further purification unless specifically mentioned. 2-Chloro-2-oxo-1,3,2-dioxaphospholane (>95%) was purchased from TCI Europe and distilled under vacuum and stored in a glove box for less than a week (substrate functionalization). Toluene, acetonitrile, diethyl ether, acetone, isopropanol, and tetrahydrofuran were purchased from Biosolve. 3-aminopropyltriethoxysilane (APTES, 99%, argon flushed) was purchased from Acros Organics. The toluene and acetonitrile used in the glove box were obtained from Fisher Scientific (anhydrous, sealed under argon). N-methylimidazole (NMI) was refluxed over sodium metal, distilled under vacuum, and stored in a glove box. Dimethyl formamide was dispensed from an MBraun SPS-800 solvent purification system equipped with aluminum oxide columns. Silicon wafers ((100), single-side polished, diameter 100 mm, thickness 525 µm) were purchased from Si-Mat. AFM tips (Bruker’s Sharp Microlever probe, MSNL-10) were purchased from Bruker.

### Synthesis

#### Sample preparation for single-molecule force spectroscopy (SMFS) experiments

Silicon wafers were cut into 1.4 by 1.4 cm squares and submerged in a beaker with acetone, which was immersed in an ultrasonic cleaning bath for 10 min. The solvent was decanted and this procedure was repeated with isopropanol and milli-Q water. The wafers were dried in a glass petri dish at 90 °C, cooled down to room temperature, and immersed in 6 mL sulfuric acid (95%). 2 mL aqueous hydrogen peroxide solution (30%) was carefully added. After 5 min the dish was placed on a hot plate at 90 °C and left for 45 min. The wafers were allowed to cool down and rinsed with de-ionized water until the water was no longer acidic, followed by three more immersions in milli-Q water. The wafers were dried under a dry flow of nitrogen gas at 90 °C overnight. Then, the wafers were immersed in a solution of APTES in dry toluene (1 mg/mL) at 50 °C for 30 min. The wafers were then immediately immersed in toluene and rinsed with toluene twice, after which they were heated under a flow of dry nitrogen gas at 90 °C for 3 h and then rinsed with acetonitrile three times. For the functionalization with polymer brushes, 1 mg HO-PEG_10k_-NHS, 9.5 mg CH_3_O-PEG_5k_-NHS and 6 µL triethylamine were dissolved in 20 mL dry acetonitrile. The wafers were immersed in this solution at room temperature for 16 h, rinsed with acetonitrile and immersed in fresh acetonitrile three times for 1 h. The wafers were dried under vacuum, transferred to a nitrogen-filled glove box, and immersed in 6 mL dry toluene. 84 mg 1-methylimidazole was added, followed by dropwise addition of 100 µL ethylene chlorophosphate. A precipitate with a gel-like consistency was formed at the bottom during the addition. After 1 h the wafers were removed, rinsed with toluene and acetonitrile, and then immersed in fresh acetonitrile twice for at least 30 min. The wafers were dried under vacuum and stored in a glove box or other nitrogen-filled container until use.

AFM tips were immersed for 20 s each in acetone, isopropanol, and milli-Q water. They were then dried under a gentle stream of dry nitrogen gas from an inverted funnel. 6 drops of sulfuric acid (95%) were added to a glass dish and the tips were carefully positioned inside the droplet. Two drops of aqueous hydrogen peroxide (30%) were carefully added, and the tips were left at room temperature for 45 min. After carefully removing the liquid, milli-Q water was added and removed five times. The tips were then immersed in milli-Q water three more times and dried under a gentle flow of dry nitrogen for 16 h at 90 °C. Amination of the tip was carried out analogous to the procedure for the silicon wafers described above. Tips were immersed in an APTES solution in toluene (1 mg/mL) at 50 °C for 30 min, followed by immersion in toluene (3 × 20 s), heating under a gentle stream of nitrogen at 90 °C, and three more immersions in acetonitrile for 20 s and gentle drying under nitrogen flow. For functionalization with polymers, the tips were immersed in a solution containing 3 mg HO-PEG_10k_-NHS and 2 mg triethylamine in 6 mL acetonitrile for 16 h. The tips were then immersed in fresh acetonitrile once for 30 min and three times for 20 s, followed by 30 s in diethyl ether. The tips were dried under a gentle flow of nitrogen and stored in a glove box or other nitrogen-filled container before use.

### Characterization

#### General

NMR spectra were recorded on a Varian Gemini or a Bruker Avance III spectrometer at 25 °C (400 MHz for ^1^H, 162 MHz for ^31^P). XPS spectra were recorded using a Thermo Scientific K-alpha spectrometer equipped with an Al Kα (17.86 eV) X-ray source. CasaXPS software was used for the analysis of the XPS data.

#### SMFS measurements

SMFS measurements were carried out on a JPK ForceRobot® 300 automated force spectroscope, using triangular cantilever A on the functionalized multi-probe tip in a fluid cell. The wafer and tip were installed and the fluid cell was filled from the side with dry DMF that was stored over 4 Å molecular sieves and filtered through a 0.2 µm PTFE syringe filter immediately prior to measuring. The syringe and filter were left connected to the fluid cell during the measurement and the exit was attached to a constricted piece of PEEK tubing to minimize the contact between air and solvent during measurements. The setup with solvent was equilibrated at 60 °C for at least 90 min prior to calibration and measurement. Calibration was performed using the thermal vibration method using the second harmonic oscillation at 38.66 kHz with a correction factor of 0.251. The second harmonic oscillation was used over the first due to viscous damping caused by the solvent. The observed spring constant of 0.073 N/m matched the spring constant indicated by the manufacturer (0.070 N/m). Information on measurement sequence and data processing can be found in the supplementary information.

#### General computational details

Calculations were carried out using the Amsterdam Density Functional suite (AMS 2021.102)^21–23^ using the OLYP functional, consisting of the optimized exchange functional^24^ and the Lee-Yang-Parr correlation functional (LYP)^25^, with a TZ2P basis set of Slater-type orbitals^27^. Scalar relativistic effects were taken into account by means of the zero-order regular approximation (ZORA)^26^. This combination of computational parameters has been found to reproduce ab initio energy barriers for S_N_2 reactions and has been used for substitution reactions on phosphate esters previously^28,29,33,34^. The numerical quality of the Becke integration grid^35,36^ was set as “VERYGOOD” and the convergence for geometric optimizations, transition state calculations, and intrinsic reaction coordinate (IRC) path calculations^37,38^ were set to 10^–5^ *E*_*h*_*/a*_0_, corresponding to 0.8 pN. Stationary points were confirmed to have either no imaginary frequencies (minima) or a single imaginary frequency (transition states) through vibrational analysis^39–41^. Structures were illustrated using CYLView^42^.

#### Force-dependent geometry and energy barrier calculations in AMS

For stressed transition state, geometry, and IRC calculations, an extensional force between the carbon atoms on the methyl groups was simulated using an external restraint with ADF’s built-in “Erf” (error function) potential add-on as described in further detail in the supplementary information.

Forces were applied to both transition states in increments of 100 pN up to 700 pN, and after that in increments of 200 pN. After every increment, the coordinates of the new transition state were used as the starting geometry for the next force. From each transition state a rough intrinsic reaction coordinate (IRC) scan of up to four steps in each direction with a step size of 0.5 √(amu∙*a*_0_) was performed, the endpoints of which were used as input for a geometric optimization to ensure that the calculated energetic minima are indeed connected by the calculated transition states.

## Supplementary information

Supplementary Information

Transparent Peer Review file

## Source data

Source Data

## Data Availability

The datasets generated during and/or analyzed during the current study are available from the corresponding author on request. Source data are provided with the manuscript. Source data are provided with this paper.

## Code availability

Matlab scripts used to analyze SMFS data are available online at https://github.com/Supramolecular-Polymer-Chemistry/CatchBondSMFS.
