# Supplementary material for: Neighbouring group participation hindered by force as a molecular design for covalent catch bonds
Source: Nat Commun. 2026 May 22;17:6751. doi: 10.1038/s41467-026-73312-9 (PMC13385849; doi:10.1038/s41467-026-73312-9)
Supplement: Supplementary file 1 — Supplementary Information [file 41467_2026_73312_MOESM1_ESM.pdf]

Supplementary Information

Neighbouring group participation hindered by force as a molecular design for covalent catch bonds

Soumabrata Majumdar,[a]† D.R. Diederik van Luijk,[a]† Martijn M. van Galen,[b,c] Pascal Vermeeren,[d] Trevor A. Hamlin,[d] F. Matthias Bickelhaupt,*[d,e,f] Joris H.B. Sprakel,[c] Rolf A.T.M. van Benthem,[g,h] Johan P.A. Heuts,[a] Rint P. Sijbesma.*[a]

Affiliations

[a] Department of Chemical Engineering & Chemistry and Institute for Complex Molecular Systems, Eindhoven University of Technology, P.O. Box 513, 5600 MB Eindhoven, The Netherlands

[b] Physical Chemistry and Soft Matter, Wageningen University and Research, Stippeneng 4, 6708 WE, Wageningen, the Netherlands

[c] Laboratory of Biochemistry, Wageningen University and Research, Stippeneng 4, 6708 WE, Wageningen, the Netherlands

[d] Department of Chemistry and Pharmaceutical Sciences, Amsterdam Institute of Molecular and Life Sciences (AIMMS), Vrije Universiteit Amsterdam, De Boelelaan 1108, 1081 HZ Amsterdam, The Netherlands

[e] Institute of Molecules and Materials (IMM), Radboud University, Heyendaalseweg 135, 6525 AJ Nijmegen, The Netherlands

[f] Department of Chemical Sciences, University of Johannesburg, Auckland Park, Johannesburg 2006, South Africa

[g] Department of Chemistry & Chemical Engineering, Laboratory of Physical Chemistry and Center for Multiscale Electron Microscopy, Eindhoven University of Technology, P.O. Box 513, 5600 MB Eindhoven, The Netherlands

[h] Energy Transition Center Amsterdam, Grasweg 31, 1031 HW Amsterdam, The Netherlands

†These authors contributed equally to this work.

*Corresponding authors: Rint P. Sijbesma, Email: r.p.sijbesma@tue.nl; F. Matthias Bickelhaupt, Email: f.m.bickelhaupt@vu.nl

Supplementary computational methods and data

Applying an external force using ADF's error function potential

The external force was simulated using the 'Erf' restraint potential. This restraint adds an external potential with magnitude:

$$V(x) = a\left(bx \cdot \operatorname{erf}(bx) + \frac{e^{-(bx)^2} - 1}{\sqrt{\pi}}\right)$$

Where *V*(*x*) is the added potential in the geometry optimization, *x* is the difference between the distance between the pulled atoms and a set distance at which the potential reaches its minimum value of 0, and a and b are parameters that can be tuned indirectly. The simulated force between the pulling points is the derivative of this potential with respect to distance, or:

$$-F(x) = \frac{dV(x)}{dx} = ab \cdot \operatorname{erf}(bx)$$

The potential *V*(*x*) resembles a V-shape with a parabolic tip that reaches a minimum at (0,0). Its derivative –*F*(*x*) converges rapidly to –*ab* for negative values of *x*, or to +*ab* for positive values of *x*. When the set interatomic distance between pulled atoms is always smaller than the setpoint, the potential closely resembles a linear downwards potential, corresponding to a constant pulling force equal to $-F_\infty = -ab$. Note that the direction of the force vector at infinite separation F_∞ is defined as attractive and thereby opposite to the direction of the force applied by the potential at separations below r_{SP}, which is therefore denoted as –*F*(*x*). For values where the interatomic distance is close to the setpoint and *x* approaches 0, the potential approaches a harmonic potential with force constant $k = 2ab^2/\sqrt{\pi}$. The relative error between the force applied by the full error force potential and the distance-independent force is given by:

$$relative\ error = \frac{-F(x) - F_\infty}{F_\infty} = \frac{ab \cdot (\operatorname{erf}(bx) + 1)}{ab} = \operatorname{erf}(bx) + 1$$

The three parameters that can be set for the restraint are $F_\infty = ab$, $k = 2ab^2/\sqrt{\pi}$, and $r_{SP} = r - x$. For these calculations *k* was set to the default value of 1 E_h/a_0^2, giving a relative error at an interatomic distance 0.1 Å below the setpoint value and at 5 nN force of 10^{-4}. In the calculations, the setpoint distance r_{sp} was set to 9.155 Å, or 4 Å above the optimized force-free initial geometry, and in none of the calculated geometries under force did the distance between loaded atoms *r* exceed (or even approach) 9.055 Å, thereby satisfying the condition $x \leq -0.1$ Å and ensuring a practically constant simulated force over the entire energy landscape even under the largest simulated forces. Under conditions where the connection between the pulling points is (unintentionally) severed, the rounded V-shape of the potential ensures that the geometry does converge with a pulling point distance equal to the set distance.

Interconversions between Int1 and Int2

The two modelled transition states do not lead to an identical intermediate structure. In assuming that the modelled transition states are the rate-limiting step of the ring-closing reaction, it must be established that the energy required to move between these transition states is negligibly low. These two intermediates are structurally related through rotation of the P–O–H bond as well as movement of the associated methanol molecule so that it maintains a hydrogen bond with this proton. Given the many possible conformations possible for such a system in general and for the explicit solvent molecule in particular, we explored the energy landscape between these two intermediates by finding geometries with two associated methanol molecules. This models a sequence in which an additional molecule of methanol associates to the first intermediate and then a second molecule of methanol

dissociates. In the experimental systems, excess alcohol functionalities are present in both the dynamic networks and in the SMFS system. However, the precise nature of the proton transfers in our experimental systems remains unknown. Functional groups likely to contribute to proton transfer, besides alcohol functionalities, include the ether and ester links in the polymer backbones, DMF in the SMFS experiments.

Four intermediate geometries with correctly rotated methyl groups were found (Supplementary Figure 1). Of those four geometries **Int 2MeOH**, displayed in the main text as an intermediate structure, is the lowest in energy and appears to be a plausible intermediate between **Int1** and **Int2**. The other three structures are close in energy and geometry and reinforce the assumption that the conformational landscape between **Int1** and **Int2** via addition of a solvent molecule is shallow compared to both transition states.

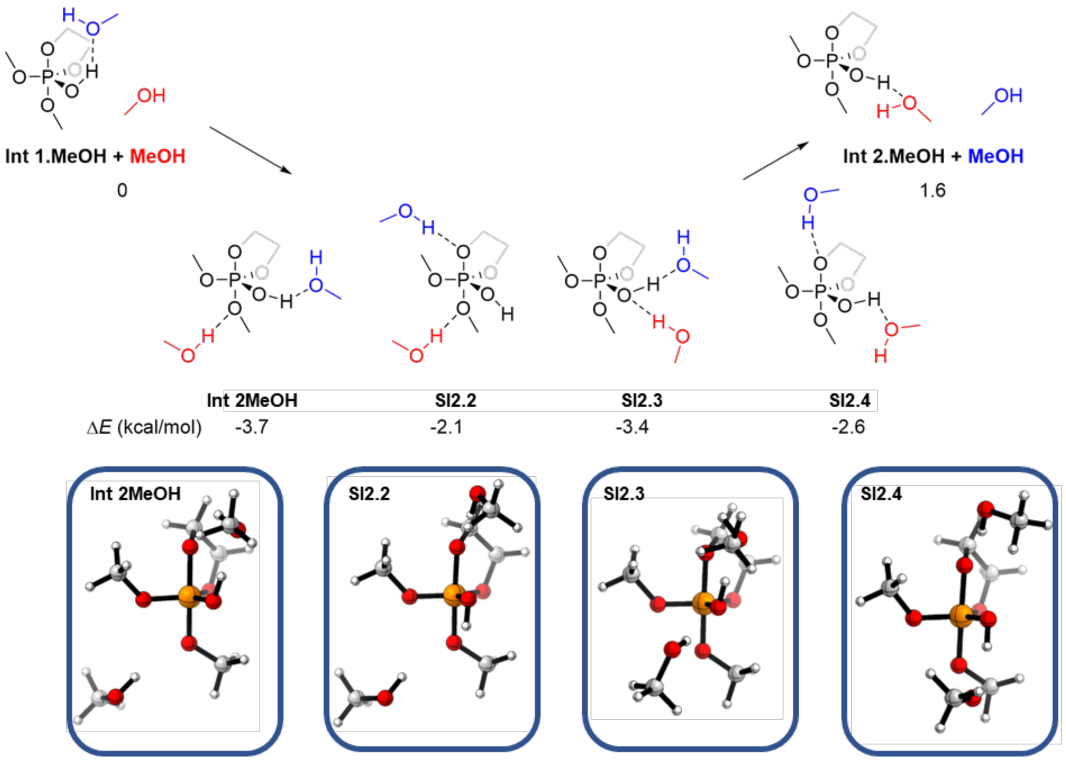

Supplementary Figure 1. Geometries of intermediates with 2 methanol molecules. Schematic structures and calculated geometries of intermediate species with 2 additional methanol molecules, with energies relative to **Int1** plus a free molecule of methanol.

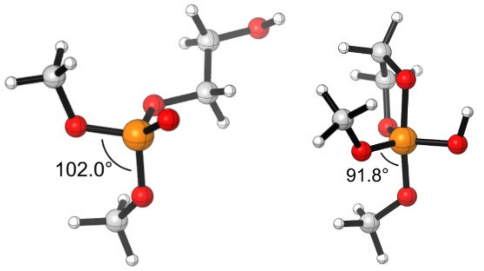

Supplementary Figure 2. OPO bond angle change during NGP transesterification in DMHEP. Calculated geometries of **DMHEP** (left) and an intermediate species (right) without explicitly modeled methanol molecules, highlighting the decrease in OPO bond angle between the methoxy groups going from a tetrahedral to a trigonal bipyramidal structure.

Using pre-rotated geometries

Stationary points were found in multiple different conformations. When analyzing the energy barriers at increasing forces starting from the lowest energy conformations, rotation of –OMe groups around the P–O bond is observed at low forces. Therefore, we selected the local minima and saddle points in a conformation where the methyl groups were already turned away from one another. These "pre-stretched" geometries were found either manually by rotating parts of the molecule and performing a new geometry optimization, or automatically from a force-loaded conformation by reducing the force and re-optimizing a new geometry in one or multiple steps.

Supplementary Table 1. Energy differences with respect to **RC** under force, calculated in 100 pN or 200 pN increments until the transition state calculation would no longer converge.

F (pN)	**RC**	**TS 1**	**Int 1**	**Int 2**	**TS 2**
	Δ*E* (kcal/mol)	Δ*E* (kcal/mol)	Δ*E* (kcal/mol)	Δ*E* (kcal/mol)	Δ*E* (kcal/mol)
0	0	30.75	21.19	19.85	33.09
100	0	30.96	21.3	21.06	34.33
200	0	31.12	21.41	22.17	35.48
300	0	31.24	21.51	23.17	36.17
400	0	31.34	21.62	23.12	36.44
500	0	31.42	21.72	23.22	36.47
600	0	31.5	21.83	23.32	36.48
700	0	31.56	21.93	23.41	36.47
900	0	31.68	22.14	23.09	36.43
1100	0	31.78	22.36	23.8	36.33
1300	0	31.89	22.59	24.01	36.19
1500	0	32	22.82	24.2	36
1700	0	32.1	23.05	24.4	35.73
1900	0	32.2	23.29	24.59	35.39
2100	0	32.3	23.52	24.77	34.93
2300	0	32.41	23.77	24.22	34.34
2500	0	32.52	24.02	24.37	33.58
2700	0	32.64	24.28	24.5	32.66
2900	0	32.75	24.53	24.61	31.64
3000	0	32.8	24.66		
3200	0	32.92	24.92		
3400	0	33.05	25.19		
3600	0	33.17	25.47		
3800	0	33.3	25.74		
4000	0	33.42	26.01		
4200	0	33.55	26.28		
4400	0	33.69	26.56		
4600	0	33.82	26.82		
4800	0	33.98	27.08		

Supplementary methods and data for SMFS experiments

Control experiment for ethylene phosphate functionalization

The reaction between ECP and alcohols is known to be catalyzed by nucleophilic nitrogen species such as NMI. The adduct of NMI and ECP is poorly soluble in apolar solvents and can undergo direct reaction with an additional nucleophile such as an aliphatic alcohol without the need to open the five-membered ring. To find suitable reaction conditions for the final surface functionalization, test reactions were performed in the glove box on 2-methoxyethanol. 4 mg 2-methoxyethanol (0.05 mmol) and 45 mg NMI (0.55 mmol) were dissolved in 0.5 mL toluene-d8. To this, 45 µL ECP was added (0.5 mmol). A gel-like precipitate immediately formed. After 10 minutes the liquid phase was sampled and measured by ^{1}H and ^{31}P NMR. (Supplementary Figure 3) Despite the tenfold excess of ECP over the alcohol, the major species in ^{31}P NMR was found to be the desired cyclic phosphate triester, indicating that most of the added ethylene chlorophosphate was converted to the precipitate. ^{1}H NMR also showed good conversion of the alcohol to the phosphate triester. Therefore, this combination should rapidly and somewhat cleanly react surface alcohol groups to the corresponding ethylene phosphate triester groups, while the excess reagents form a precipitate from which the sample is easily removed. The precipitate dissolved rapidly in acetonitrile, which was used in subsequent washing steps.

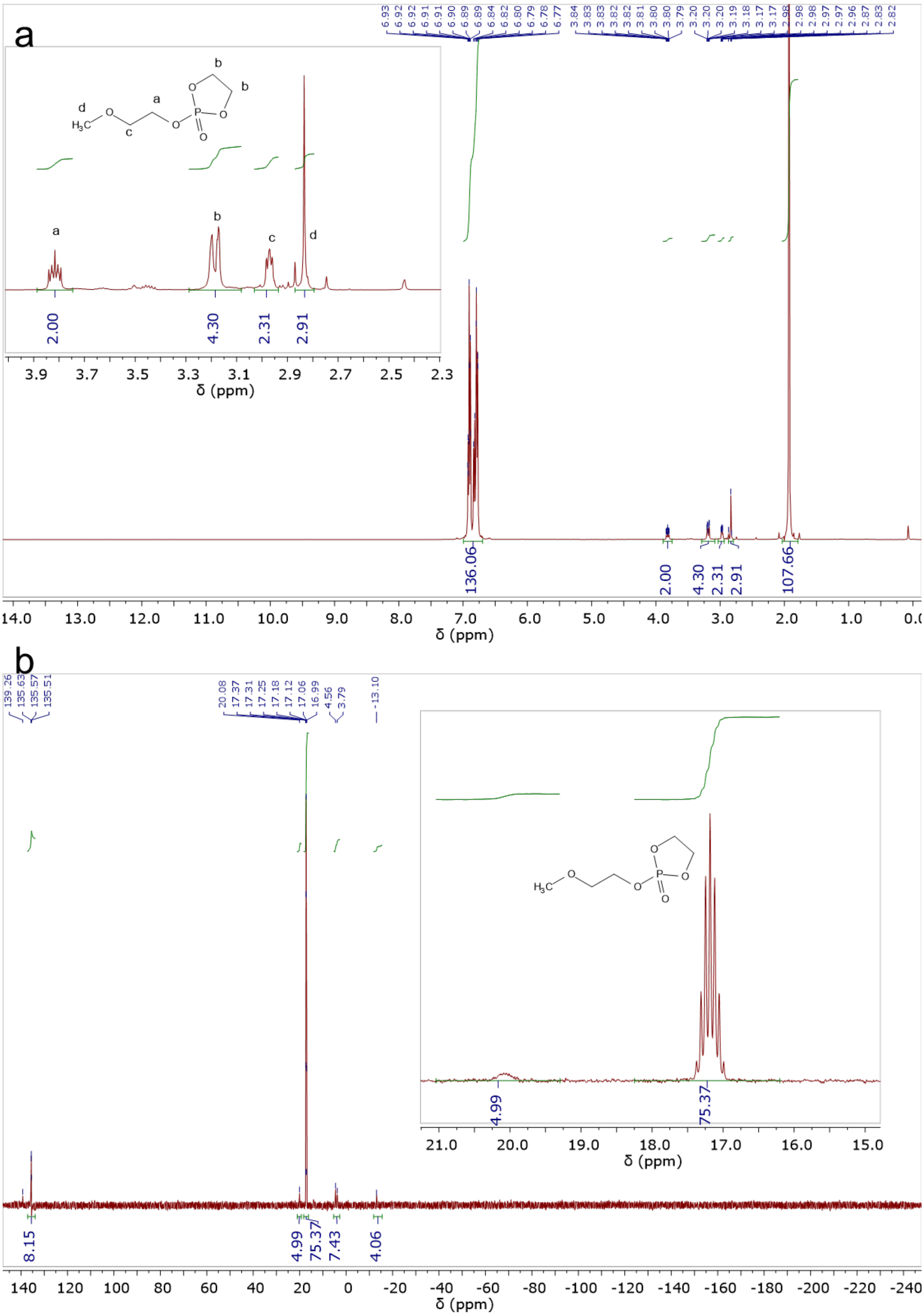

Supplementary Figure 3. Crude NMR spectra of control experiment for ethylene phosphate functionalization. a) ^{1}H NMR and b) ^{31}P NMR spectra of crude reaction mixture in toluene-d8 with zoom in insets. Integrals in b) add to 100.

SMFS measurement sequence

Each measurement comprised six blocks within a piezo Z-range of 3 µm. First, the tip was set to approach the surface until a constant force compressive force, typically set to 50 pN, was reached (**Block 1**). In **Block 2**, this compressive force was kept constant for a specific time, typically 0.5 s. These values can be adjusted to tune the average number of single-bonding events, as variation in the likeliness of bond formation can occur between batches, during the experiment (possibly due to side reactions on the sample such as hydrolysis) and due to experimental artifacts such as a force-height baseline tilt. In the **Block 3**, the tip was retracted until a constant set force was reached. This force can differ from the measured force in the experiment due to an observed dependence of the force as a function of height, which was corrected for (*vide infra*). If the force could not be reached, the experiment was characterized as a 'no-bond' measurement. If a constant tensile force could be achieved, the force was kept constant for 30 seconds. When a sufficient tensile force cannot be achieved, either due to rupture of a connection or its absence, the tip moves to the maximum of the piezo Z-range and the measurement is finished. Otherwise, after 30 seconds the tip is moved away from the surface to break any remaining connections (**Block 5**) and then held for 0.5 s to end a full measurement cycle (**Block 6**). See Supplementary Figure 4 for a schematic representation and examples.

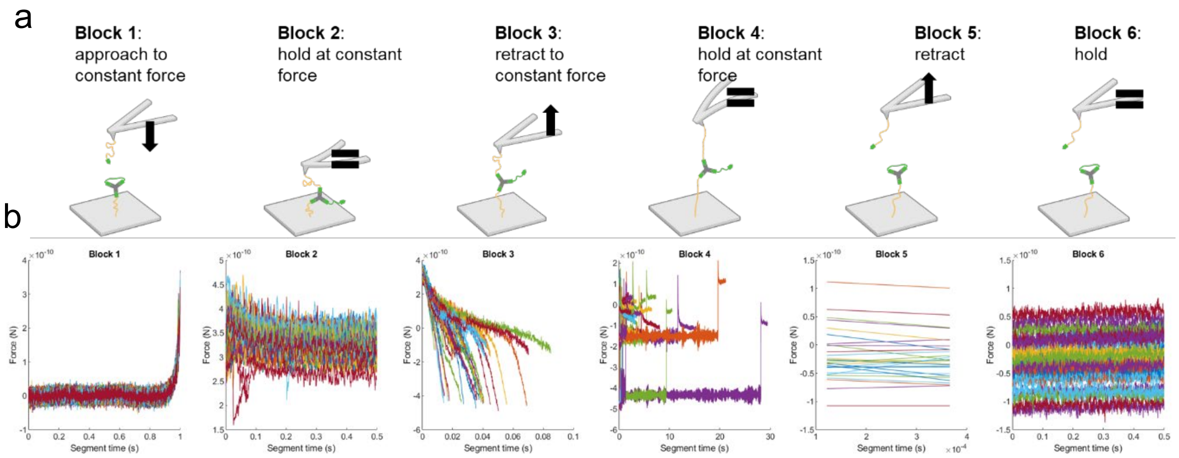

Supplementary Figure 4. Force measured at different points of the SMFS measurement sequence. Schematic overview of SMFS measurement sequence and example curves. a) Schematic overview of all 6 blocks that comprise an SMFS measurement cycle, as described in detail in the supplementary information. b) Force-time curves of a small subsection of valid events after baseline correction of **Block 1**. Even though forces were set to the same value, the drift on approach to the surface causes a distribution in measured restoring forces. Cartoons of wafers and AFM tips were made by the ICMS Animation Studio.

Choosing a minimum event time for SMFS experiments

Bond breaking events observed over 30 seconds were found to vary in lifetime from under 10^{-2} to over 20 seconds. We decided to exclude very short lifetimes for the following reason. First, it is unlikely that these events correspond to breaking a covalent bond that is formed very rapidly in situ. Given the broad distribution in lifetimes without correcting for this, it appeared that at least two different types of processes were responsible for disconnecting the tip from the substrate. The shorter population of events appeared to be more prevalent at lower forces. An unknown covalent interaction could be responsible for one of these unwanted binding event types, including electrostatic forces or chain adsorption. Due to the nature of the surface composition, partial hydrolysis to charged species can be expected in the measurement setup. Secondly, the force is applied in a retraction step immediately

prior to the main measurement. Although this step is very fast (typically between 0.01 and 0.1 seconds) it is not negligible when the bond lifetime at constant force is far below 1 s. This introduces uncertainty in both the lifetime and the applied force. Additionally, for bonds that break almost instantaneously the sampling rate used (546 Hz) is not sufficient to accurately determine the timepoint of breaking.

We therefore chose a cutoff time before which the majority of “very fast” events had already occurred and that longer than the typical retraction time, while maintaining events that could plausibly be caused by a covalent event. Based on the observed distributions in Supplementary Figure 5b and 5c we selected a time of 0.2 s.

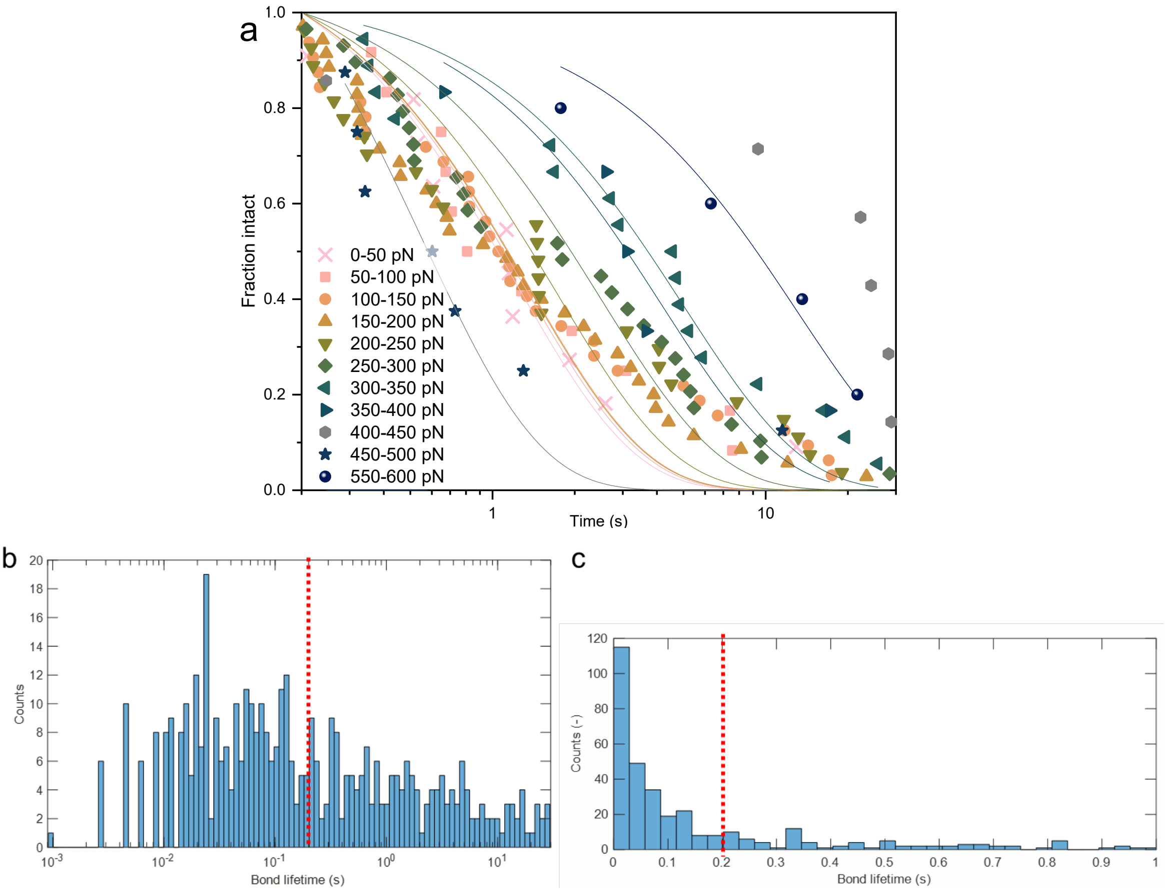

Supplementary Figure 5. Fitted curves and parameters for SMFS experiments. a) Data points for events in all force bins and exponential decay fits used to extract a characteristic lifetime. b) logarithmic histogram of single events in all force bins, including points that were considered to break too early; c) linear histogram of all events in the first second. The cutoff applied in order to remove very rapid bond breaking events at $t_{CO} = 0.2$ s is given by the red dashed line.

SMFS data processing and analysis

For each measurement, all blocks were important and analyzed with a custom Matlab script. Each measurement was sorted into one of the following categories: 'no-bond' when no connection was formed as found by the absence of a restoring force in the third ‘retraction’ block; 'multi-bond' when a jump in height was observed of less than 15 nm and the applied force was restored before a second break occurred during or after measurement, or when multiple peaks in the force-height diagram were observed in the retraction after the constant-force measurement; 'single bond broken in

retraction' when no event was found during the measurement time but a single disconnection was found after; 'other or unknown' when interpretation of the force curves was ambiguous.

Because the measured force was found to have a linear deviation with the measured height above the surface, a baseline correction was then performed. Of the first block (approach to the surface), the first 90% of the force-height curve was fitted to a straight line which was subtracted from all blocks in measurements.

For finding events, we initially attempted to automatically extract an event time and corresponding restoring force. The height-time curve in the fourth block (constant tensile force) was smoothed and the largest positive second derivative was selected as the location of the event. Force was averaged over up to 10 datapoints before and after detected event to extract the restoring force. The automatically extracted position was checked for each event manually. Forces before and after could be manually selected if event finding did not agree with the visually identified position.

Events from two individual datasets were combined and sorted into bins with a width of 50 pN bins. Events with a lifetime under 0.2 s were removed to correct for highly short-lived, likely noncovalent, interactions. Only force bins with at least 3 datapoints were considered. At each time where a connection was severed, the fraction intact, starting from 1–1/(N+1), was reduced by 1/(N+1), where N is the total number of events in the force bin. These decay curves were fitted to an exponential decay curve normalized to 1 at the cutoff time t_{CO} = 0.2 s according to Supplementary Equation 1, and can be found in Supplementary Figure 5a.

$$f = e^{-(t-t_{CO})/\tau} \qquad \text{(Supplementary Equation 1)}$$

Where f is the fraction of connection between AFM tip and wafer that is still intact as a function of time t, t_{CO} is the cutoff time below which events are not included (here 0.2 s), and τ is the characteristic lifetime.

Supplementary Table 2. Fit parameters for each force bin in the SMFS experiments from fitting the decay plots in Supplementary Figure 5a.

Force bin (pN)	Number of data points	τ (s)	Standard error (s)	Reduced χ^2
0–50	10	1.22	0.096	0.94
50–100	11	1.30	0.154	0.89
100–150	31	1.36	0.096	0.90
150–200	34	1.35	0.117	0.86
200–250	26	1.72	0.216	0.78
250–300	28	2.51	0.228	0.86
300–350	17	5.05	0.396	0.92
350–400	5	4.19	0.755	0.83
400–450[1]	6	*n.d.*	*n.d.*	
450-500	7	0.55	0.081	0.88
550–600	4	13.08	1.306	0.95

[1]No suitable fitting parameters found

XPS spectra after functionalization steps

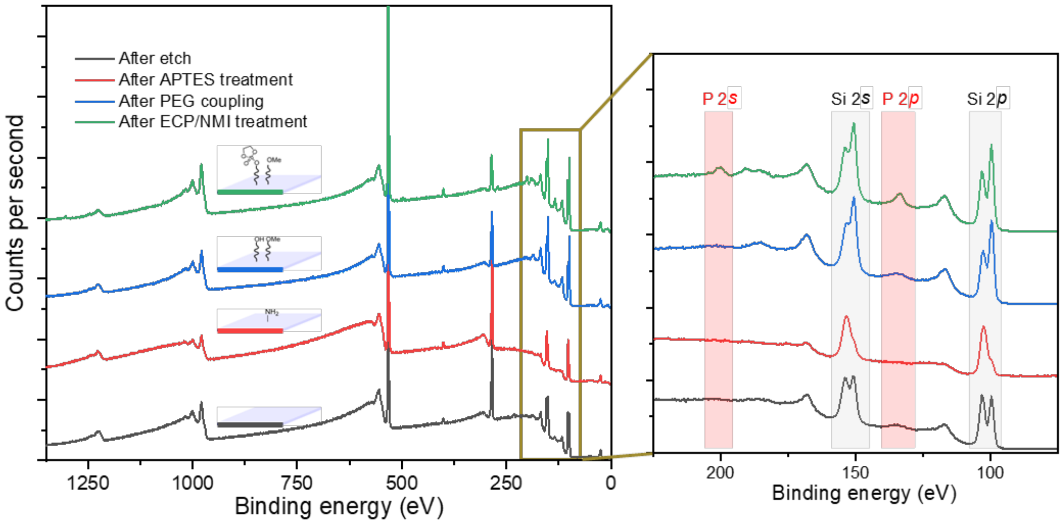

Supplementary Figure 6: **XPS spectra after each treatment step.** The P 2s band was found to be useful for characterizing the final step, as the P 2p band had more overlap with Si 2p plasmon resonance bands from the substrate.

SMFS results as a scatter plot

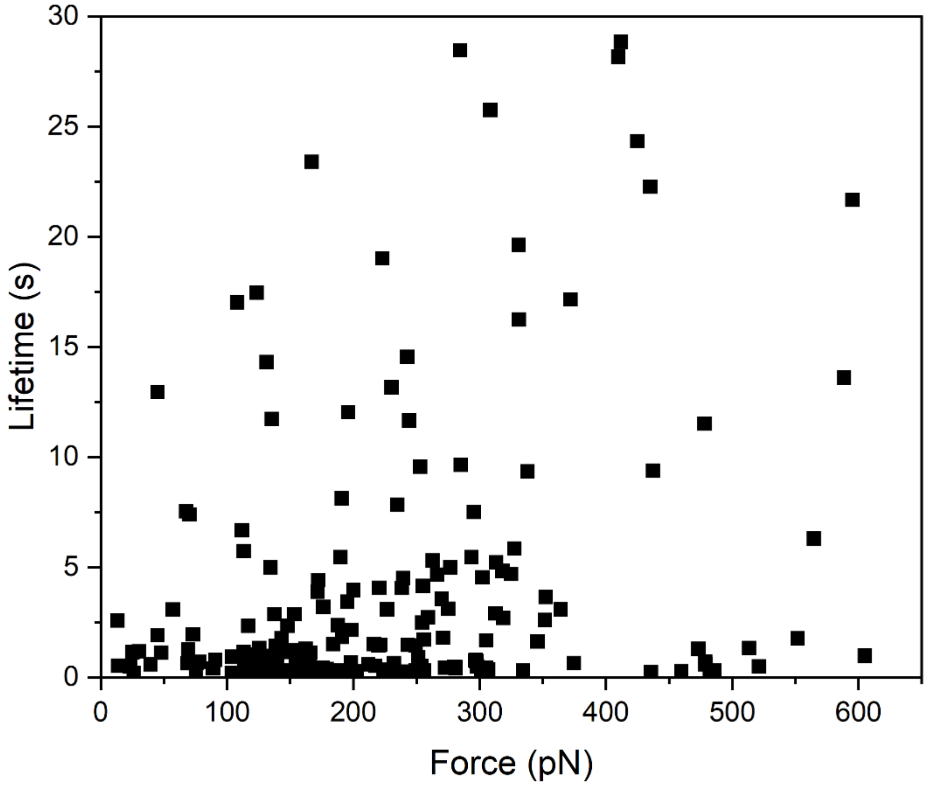

Supplementary Figure 7: **All valid force-lifetime datapoints from SMFS measurements** with a lifetime over 0.2 s represented as a scatter plot.

SMFS results with 25 pN force interval

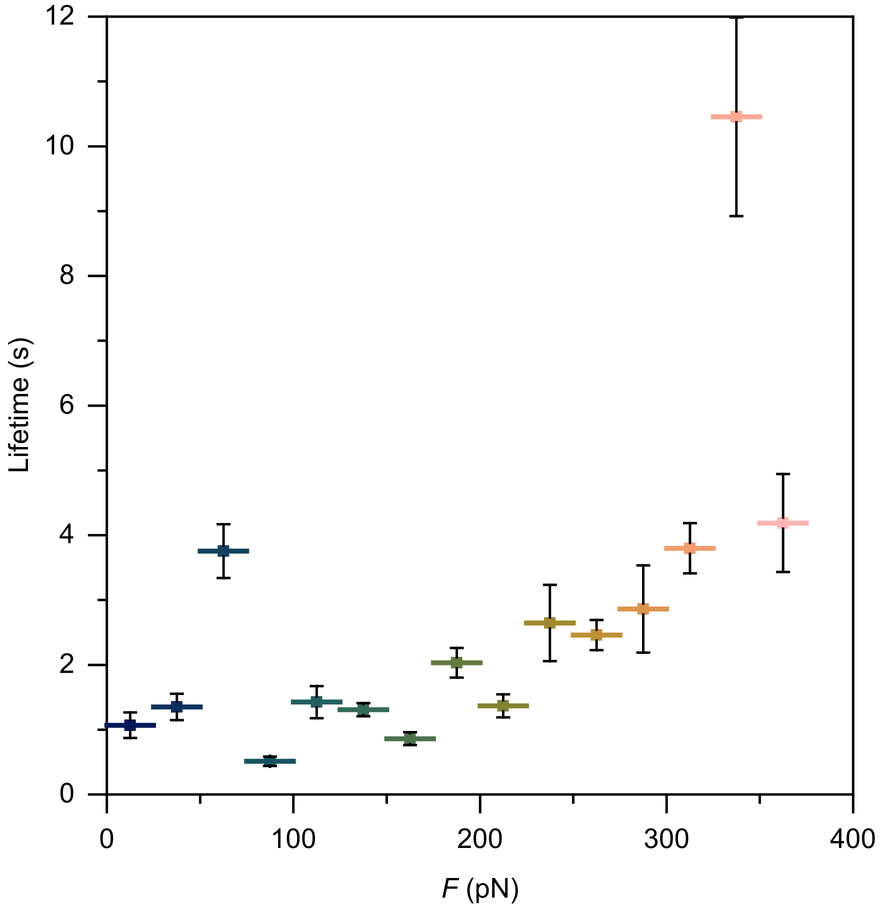

Supplementary Figure 8: **SMFS results with a 25 pN force interval** showing a similar trend of increasing lifetimes between 200 and 400 pN. Error bars represent ± one standard deviation.
