# Supplementary material for: Neighbouring group participation hindered by force as a molecular design for covalent catch bonds
Source: Nat Commun. 2026 May 22;17:6751. doi: 10.1038/s41467-026-73312-9 (PMC13385849; doi:10.1038/s41467-026-73312-9)
Supplement: Supplementary file 2 — Transparent Peer Review file [file 41467_2026_73312_MOESM2_ESM.pdf]

nature portfolio

Peer Review File

Neighbouring group participation hindered by force as a molecular design for covalent catch bonds

Corresponding Author: Professor Rint Sijbesma

This manuscript has been previously reviewed at another journal. This document only contains information relating to versions considered at Nature Communications.

This file contains all reviewer reports in order by version, followed by all author rebuttals in order by version.

Version 0:

Reviewer comments:

Reviewer #2

(Remarks to the Author)
Thank you for your thoughtful and transparent response to the reviewers' comments. I appreciate the authors' willingness to critically reassess their earlier interpretation and to revise the manuscript accordingly. In particular, I commend the authors for taking the reviewers' feedback seriously and for using it constructively to strengthen the work.

The decision to remove the rheology and network solvolysis results improves the rigor and credibility of the work. Refocusing the study on the DFT and SMFS results also provides a clearer and more compelling foundation for the revised claims.

Overall, the revised manuscript is well-written, and the conclusion that hydroxyethyl triphosphate (HEP) esters behave as catch-bonds due to neighboring group participation (NGB) appears to be supported by the DFT calculations and SMFS measurements. However, I have several additional comments that I believe merit consideration before publication. Specifically,

1. Abstract (Lines 40–43): The authors state that the NGP principle can enable the "design of force-responsive polymer networks with tunable mechanical properties." Because the revised manuscript no longer presents data on bulk polymer networks, I do not think this claim is sufficiently supported in its current form and should therefore be removed or substantially tempered.

2. Lines 66–70: The authors state that they "propose a general molecular mechanism for covalent catch bonding when bond dissociation takes place via NGP." At present, however, the study appears to focus exclusively on the HEP motif, which makes the breadth of this claim difficult to assess. Unless the authors can provide evidence that this behavior extends to an additional chemical system, I encourage them to qualify this statement and more clearly frame the proposed mechanism in the context of the specific chemistry examined here.

3. Line 123: The authors refer to Supplementary Table 4, but I was unable to locate this table in the Supplementary Information. Did the authors intend to refer instead to Supplementary Table 2? Please clarify and correct the reference as needed.

4. Lines 145-146: The authors state that, following reformation of the five-membered ring and loss of connection between the tip and the wafer, there is "a sudden loss of tensile force and an increase in tip height to restore the force." I find this description difficult to follow. Once the connection between the tip and the wafer is lost, I would have expected the measured force to drop to zero. Could the authors clarify the physical basis by which the force is restored in this scenario and explain more explicitly what is meant by the increase in tip height restoring the force?

5. Lines 151-152: The authors state that the "lifetimes are roughly constant up to a force of 200 pN and increase to approximately 4 s at 400 pN." If I understand the analysis correctly, this trend appears broadly consistent with the DFT results, which predict an increase in activation energy from 35.48 to 36.44 kcal mol^{-1}. Assuming an Arrhenius-type

interpretation, this change would correspond to an approximately 5-fold increase in lifetime at 298 K. The authors may wish to highlight this quantitative consistency more explicitly in the revised manuscript, as it would help strengthen the connection between the DFT calculations and the SMFS measurements.

Reviewer #3

(Remarks to the Author)
The manuscript has been substantially improved in the revised version. The authors openness and honesty about the in material effects of their hypothesis is appreciated, and the molecular characterization and simulation of this novel phenomonen still support publication in Nature Communications. I support publication without further revisions.

Reviewer #4

(Remarks to the Author)
[Note from editor: Reviewer #4 was asked to comment on the authors' responses to Reviewer #1's concerns]

Reviewer #1 raised a series of questions regarding the previous draft of the manuscript. Specifically:
• The reviewer questioned whether covalent catch bonds are the sole cause of the material's unique macroscopic mechanical properties, as these properties could be influenced by other factors such as viscoelasticity, poroelasticity, hydrophilicity and hydrophobicity.
• It was unclear whether the individual HEP ester bonds in the macroscopic tensile experiments truly exhibited catch-bond behavior as opposed to slip.
• The potential influence of loading rate on the material's constitutive behavior needs to be further explored.
• The quantity of experimental samples is insufficient.
These points of contention were primarily directed at the macroscopic experimental results. During the revision process, the authors acknowledged that the interpretation of the macroscopic polymer network experiments could be confounded by interfering factors such as clamp relaxation effects and complex stress fields. While the macroscopic manifestation of the force-hindered neighboring group participation reaction observed at the single-molecule level in HEP triesters may be challenging to isolate and demonstrate in polymer networks, this could be a fruitful avenue for future work. To avoid controversy surrounding these macroscopic experiments, the authors have chosen to remove the related, contentious tensile and rheological data from the revised manuscript. Consequently, the claim regarding the presence of a catch-bond effect in bulk polymer networks has been retracted.
In response to these substantial revisions, the authors have now refocused the manuscript on the molecular-level evidence, providing a more in-depth and robust presentation of the force-hindered neighboring group participation reaction (covalent catch-bond character) in HEP triesters. This is achieved through comprehensive DFT calculations and SMFS experiments. The shift in focus provides more comprehensive and reliable evidence, significantly enhancing the credibility of the conclusions. However, in my view, while the authors have provided a well-reasoned and scientifically rigorous response, the current manuscript with the removal of the macroscopic experiments relies solely on DFT calculations and SMFS experiments, which may not fully reflect the breadth of the work originally undertaken. It remains unclear how the observed NGP of HEP would affect the properties in bulk materials.

Open Access This Peer Review File is licensed under a Creative Commons Attribution 4.0 International License, which permits use, sharing, adaptation, distribution and reproduction in any medium or format, as long as you give appropriate credit to the original author(s) and the source, provide a link to the Creative Commons license, and indicate if changes were made.
In cases where reviewers are anonymous, credit should be given to 'Anonymous Referee' and the source.
The images or other third party material in this Peer Review File are included in the article's Creative Commons license, unless indicated otherwise in a credit line to the material. If material is not included in the article's Creative Commons license and your intended use is not permitted by statutory regulation or exceeds the permitted use, you will need to obtain permission directly from the copyright holder.
To view a copy of this license, visit https://creativecommons.org/licenses/by/4.0/

ANSWER TO REVIEWERS' COMMENTS

Reviewer #2 (Remarks to the Author):

Thank you for your thoughtful and transparent response to the reviewers' comments. I appreciate the authors' willingness to critically reassess their earlier interpretation and to revise the manuscript accordingly. In particular, I commend the authors for taking the reviewers' feedback seriously and for using it constructively to strengthen the work.

The decision to remove the rheology and network solvolysis results improves the rigor and credibility of the work. Refocusing the study on the DFT and SMFS results also provides a clearer and more compelling foundation for the revised claims.

Overall, the revised manuscript is well-written, and the conclusion that hydroxyethyl triphosphate (HEP) esters behave as catch-bonds due to neighboring group participation (NGB) appears to be supported by the DFT calculations and SMFS measurements. However, I have several additional comments that I believe merit consideration before publication. Specifically,
We thank the reviewer for the positive response on the new version of the manuscript. We have considered the additional comments from the reviewer and have revised the manuscript further. The comments have been addressed below.

1. Abstract (Lines 40–43): The authors state that the NGP principle can enable the "design of force-responsive polymer networks with tunable mechanical properties." Because the revised manuscript no longer presents data on bulk polymer networks, I do not think this claim is sufficiently supported in its current form and should therefore be removed or substantially tempered.

The above-mentioned statement was intended to emphasize the prospects of the reported catch bonding mechanism for the design of force responsive polymer networks. To address the reviewer's comment, we have softened the statement: "*This work establishes a molecular mechanism for engineering covalent catch bonds, offering opportunities to design force-responsive polymer networks.*"

2. Lines 66–70: The authors state that they "propose a general molecular mechanism for covalent catch bonding when bond dissociation takes place via NGP." At present, however, the study appears to focus exclusively on the HEP motif, which makes the breadth of this claim difficult to assess. Unless the authors can provide evidence that this behavior extends to an additional chemical system, I encourage them to qualify this statement and more clearly frame the proposed mechanism in the context of the specific chemistry examined here.

To address the reviewer's comments, the word "*general*" has been removed from the statement and in the subsequent statement, the mention of generalizability has been softened to a promising future prospect: "*We validate this concept using a hydroxyethyl phosphate (HEP) triester linkage*

embedded in a polymer and suggest that the approach holds promise to be generalized to other NGP-based systems with appropriate modifications."

Additionally, we would like to highlight a manuscript that is in preparation, a pre-print of which has recently been published: https://doi.org/10.48550/arXiv.2603.09911 . This pre-print uses molecular dynamics simulations to investigate force-disrupted neighboring group participation in dynamically crosslinked hydrogels.

3. Line 123: The authors refer to Supplementary Table 4, but I was unable to locate this table in the Supplementary Information. Did the authors intend to refer instead to Supplementary Table 2? Please clarify and correct the reference as needed.

We indeed meant to refer to what was at the time Supplementary Table 2 (now Supplementary Table 1) and are grateful for the opportunity to correct this mistake.

4. Lines 145-146: The authors state that, following reformation of the five-membered ring and loss of connection between the tip and the wafer, there is "a sudden loss of tensile force and an increase in tip height to restore the force." I find this description difficult to follow. Once the connection between the tip and the wafer is lost, I would have expected the measured force to drop to zero. Could the authors clarify the physical basis by which the force is restored in this scenario and explain more explicitly what is meant by the increase in tip height restoring the force?

The measured force does indeed drop to zero, but as the machine is trying to maintain a constant nonzero force the feedback mechanism moves the AFM tip up. If the connection is indeed lost, this does not actually restore the force but just keeps the tip moving to the top end of the measurement range.

To prevent confusion without resorting to a detailed description of the details of the tip position control, we decided to remove the second part of the sentence, and simply state: *" This is observed as a sudden loss of tensile force and an increase in tip height."* Readers interested in the details of the experiment may find these in the supplementary information.

5. Lines 151-152: The authors state that the "lifetimes are roughly constant up to a force of 200 pN and increase to approximately 4 s at 400 pN." If I understand the analysis correctly, this trend appears broadly consistent with the DFT results, which predict an increase in activation energy from 35.48 to 36.44 kcal mol^{-1}. Assuming an Arrhenius-type interpretation, this change would correspond to an approximately 5-fold increase in lifetime at 298 K. The authors may wish to highlight this quantitative consistency more explicitly in the revised manuscript, as it would help strengthen the connection between the DFT calculations and the SMFS measurements.

We have hesitated to quantitatively compare the DFT calculations and SMFS measurements in too much detail, particularly due to their vastly different environments (vacuum with assistance of one or two molecules of methanol or DMF at 333 K, respectively) and their associated uncertainties. Perhaps this was an overabundance of caution. We have modified the statement to:

"This finding is qualitatively supported by single-molecule force spectroscopy, which shows a more than threefold increase in bond lifetime between 200 and 400 pN."

Reviewer #3 (Remarks to the Author):

The manuscript has been substantially improved in the revised version. The authors openness and honesty about the in material effects of their hypothesis is appreciated, and the molecular characterization and simulation of this novel phenomenon still support publication in Nature Communications. I support publication without further revisions.
The authors thank the reviewer for the positive response to the new version of the manuscript.

Reviewer #4 (Remarks to the Author):

[Note from editor: Reviewer #4 was asked to comment on the authors' responses to Reviewer #1's concerns]

Reviewer #1 raised a series of questions regarding the previous draft of the manuscript. Specifically:
• The reviewer questioned whether covalent catch bonds are the sole cause of the material's unique macroscopic mechanical properties, as these properties could be influenced by other factors such as viscoelasticity, poroelasticity, hydrophilicity and hydrophobicity.
• It was unclear whether the individual HEP ester bonds in the macroscopic tensile experiments truly exhibited catch-bond behavior as opposed to slip.
• The potential influence of loading rate on the material's constitutive behavior needs to be further explored.
• The quantity of experimental samples is insufficient.
These points of contention were primarily directed at the macroscopic experimental results. During the revision process, the authors acknowledged that the interpretation of the macroscopic polymer network experiments could be confounded by interfering factors such as clamp relaxation effects and complex stress fields. While the macroscopic manifestation of the force-hindered neighboring group participation reaction observed at the single-molecule level in HEP triesters may be challenging to isolate and demonstrate in polymer networks, this could be a fruitful avenue for future work. To avoid controversy surrounding these macroscopic experiments, the authors have chosen to remove the related, contentious tensile and rheological data from the revised manuscript. Consequently, the claim regarding the presence of a catch-bond effect in bulk polymer networks has been retracted.
In response to these substantial revisions, the authors have now refocused the manuscript on the molecular-level evidence, providing a more in-depth and robust presentation of the force-hindered neighboring group participation reaction (covalent catch-bond character) in HEP triesters. This is achieved through comprehensive DFT calculations and SMFS experiments. The shift in focus provides more comprehensive and reliable evidence, significantly enhancing the credibility of the conclusions. However, in my view, while the authors have provided a well-

reasoned and scientifically rigorous response, the current manuscript with the removal of the macroscopic experiments relies solely on DFT calculations and SMFS experiments, which may not fully reflect the breadth of the work originally undertaken. It remains unclear how the observed NGP of HEP would affect the properties in bulk materials.

We thank the reviewer for appreciating the scientific rigor in the DFT and SMFS study that was used to prove the catch bond effect in HEP triester. The material aspect of the original work, which we decided to remove from the current manuscript, is further being investigated to better understand the effect of catch bonding in bulk polymer materials.
